# Supplementary material for: Helicobacter pylori eradication improves motor fluctuations in advanced Parkinson’s disease patients: A prospective cohort study (HP-PD trial)
Source: PLoS One. 2021 May 4;16(5):e0251042. doi: 10.1371/journal.pone.0251042 (PMC8096108; doi:10.1371/journal.pone.0251042)
Supplement: S1 Table — (DOCX) [file pone.0251042.s002.docx]

**S1 Table. Clinical symptoms before and after successful *Helicobacter pylori* (HP) eradication with corrected *p*-values (*n* = 17).**

|  | **Before HP eradication** | **After HP eradication** | **Corrected  *p*-value^a^** |
| --- | --- | --- | --- |
| Total TWOQ-9 score | 6.0 ± 1.3 | 4.4 ± 1.7 | **0.029** |
| - Tremor | 0.8 ± 0.4 | 0.5 ± 0.5 | 0.261 |
| - Mood changes | 0.4 ± 0.5 | 0.1 ± 0.2 | 0.841 |
| Total GI symptom score | 12.8 ± 7.3 | 8.1 ± 4.5 | 0.203 |
| - Bloating | 2.9 ± 3.2 | 1.1 ± 1.6 | 0.203 |
| - Dysphagia | 0.7 ± 1.0 | 0.3 ± 0.6 | 0.841 |
| PDQ-8 score | 10.9 ± 5.3 | 8.6 ± 5.2 | 1.000 |
| L-Dopa onset time (min) | 21.2 ± 6.9 | 19.5 ± 9.6 | 1.000 |
| L-Dopa peak time (min) | 35.7 ± 7.9 | 34.9 ± 13.8 | 1.000 |
| Daily ‘on’ time (h) | 10.9 ± 1.9 | 11.8 ± 2.0 | 0.261 |
| Daily ‘off’ time (h) | 4.7 ± 1.5 | 4.0 ± 2.1 | 1.000 |
| Dyskinesia (h) | 1.1 ± 1.6 | 1.3 ± 1.8 | 1.000 |

^a^ Bonferroni adjustment

TWOQ-9: Thai version of the 9-item Wearing-off Questionnaire; GI: gastrointestinal; PDQ: Parkinson’s Disease Questionnaire; L-Dopa: levodopa

Statistically significant *p*-values are in bold
