# Supplementary material for: Helicobacter pylori eradication improves motor fluctuations in advanced Parkinson’s disease patients: A prospective cohort study (HP-PD trial)
Source: PLoS One. 2021 May 4;16(5):e0251042. doi: 10.1371/journal.pone.0251042 (PMC8096108; doi:10.1371/journal.pone.0251042)
Supplement: S1 Protocol — (PDF) [file pone.0251042.s003.pdf]

แบบเสนอโครงการวิจัย

(ดัดแปลงจากแบบเสนอโครงการวิจัยเพื่อขอการรับรอง วันที่ 20 กันยายน พ.ศ.2561)

ผลการกำจัดเชื้อเฮลิโคแบคเตอร์ ไพโลไร ต่อการตอบสนองต่อยาไม่สม่ำเสมอในผู้ป่วยพาร์กินสัน

ชื่อหัวหน้าโครงการวิจัย

รองศาสตราจารย์ นายแพทย์ประวีณ โล่ห์เลขา

ผู้วิจัยร่วม

นายแพทย์ธนาคาร ศรีพนม

ศาสตราจารย์ ดร. นายแพทย์รัฐกร วิไลชนม์

ภาควิชาอายุรศาสตร์ คณะแพทยศาสตร์ มหาวิทยาลัยธรรมศาสตร์

95 หมู่ 8 ถนน พหลโยธิน อำเภอคลองหลวง

จังหวัดปทุมธานี 12120 ประเทศไทย

หลักการและเหตุผลที่ต้องทำวิจัย

โรคพาร์กินสันเป็นหนึ่งในโรคที่เกิดจากความเสื่อมทางระบบประสาทที่พบได้บ่อยในผู้สูงอายุ จากการศึกษาในประเทศไทยพบความชุกของโรคพาร์กินสันประมาณ 424.57 รายต่อประชากร 100,000 คน แม้ว่าในปัจจุบันจะมีการพัฒนาความรู้เกี่ยวกับการรักษาโรคพาร์กินสันเป็นอย่างมาก แต่ยาลีโวโดปายังจัดเป็นยาที่ได้ผลดีที่สุดในการบรรเทาอาการ ผู้ป่วยพาร์กินสันเกือบทุกรายจะต้องได้รับการรักษาด้วยยาลีโวโดปาเพื่อควบคุมอาการทางการเคลื่อนไหวและควบคุมคุณภาพชีวิต อย่างไรก็ตามการรักษาด้วยยาลีโวโดปาเป็นเวลานานในผู้ป่วยพาร์กินสันจะมีความสัมพันธ์กับการเกิดผลข้างเคียงทางการเคลื่อนไหวจากการรักษา ได้แก่ อาการตอบสนองต่อยาไม่สม่ำเสมอ และอาการยุกยิก ภายหลังจากการรักษาด้วยยาลีโวโดปาประมาณ 5 ปี ผู้ป่วยมากกว่าครึ่งหนึ่งจะเริ่มมีผลข้างเคียงดังกล่าวซึ่งส่งผลต่อการใช้ชีวิตประจำวันและคุณภาพชีวิต แม้ว่าโรคพาร์กินสันจะเป็นโรคที่ทำให้เกิดอาการทางการเคลื่อนไหวเป็นหลัก แต่เป็นที่ยอมรับกันว่าอาการที่นอกเหนือการเคลื่อนไหวก็สามารถพบร่วมได้มาก อาการทางระบบทางเดินอาหารเป็นอาการหนึ่งซึ่งเป็นปัญหาหลักในผู้ป่วยพาร์กินสันและอาจนำมาก่อนอาการทางการเคลื่อนไหวได้หลายปี จากหลักฐานในปัจจุบันเชื่อว่าการเปลี่ยนแปลงของแบคทีเรียขนาดเล็กน ลำไส้และสมองมีส่วนในการเกิดพยาธิกำเนิดและการดำเนินโรคพาร์กินสัน นอกจากนี้ยังพบว่าผู้ป่วยที่มีอาการท้องผูกเรื้อรังหรือลำไส้อักเสบมีความเสี่ยงต่อการเกิดโรคพาร์กินสันที่เพิ่มขึ้น

เชื้อเฮลิโคแบคเตอร์ ไพโลไร หรือเอชพี เป็นแบคทีเรียชนิดแกรมลบที่เป็นสาเหตุทำให้เกิดการอักเสบในกระเพาะอาหารและลำไส้ ซึ่งมีความสัมพันธ์กับโรคทางระบบทางเดินอาหารหลายโรค เช่น โรคกระเพาะอาหาร โรคลำไส้อักเสบ และโรคมะเร็งกระเพาะอาหาร เป็นต้น ความชุกของการติดเชื้อเอชพีในประชากรไทยพบได้ประมาณ ร้อยละ 45.9 โดยเพศชายมีความเสี่ยงมากกว่าเพศหญิง อัตราการติดเชื้อในทุกช่วงอายุพบโดยเฉลี่ยประมาณร้อยละ 40-50 และมีอัตราการติดเชื้อสูงสุดที่ร้อยละ 49.3 ในกลุ่มประชากรวัยกลางคนหรืออายุประมาณ 40-49 ปี จากการศึกษาขนาดใหญ่พบว่าผู้ป่วยที่มีการติดเชื้อเอชพี เรื้อรังจะมีความเสี่ยงต่อการเกิดโรคพาร์กินสันที่เพิ่มขึ้น และยังพบความชุกของการติดเชื้อเอชพีในผู้ป่วยพาร์กินสันมากกว่าประชากรทั่วไป นอกจากนี้ผู้ป่วยพาร์กินสันที่มีการติดเชื้อเอชพีมักมีอาการทางการเคลื่อนไหวที่แย่กว่า เนื่องจากปัญหาทางระบบทางเดินอาหารอาจส่งผลต่อการดูดซึมและระดับยาลีโวโดปา ส่งผลให้เกิดการตอบสนองต่อยาที่ไม่สม่ำเสมอ ดังนั้นการกำจัดเชื้อเอชพีจึงน่าจะส่งผลในทางบวกแก่ผู้ป่วยพาร์กินสัน

วัตถุประสงค์ของการวิจัย

วัตถุประสงค์หลัก

- เพื่อศึกษาผลของการรักษาการติดเชื้อเอชพีต่อการตอบสนองต่อยาไม่สม่ำเสมอในผู้ป่วยพาร์กินสัน

วัตถุประสงค์รอง

- เพื่อศึกษาผลของการรักษาการติดเชื้อเอชพีต่ออาการทางการเคลื่อนไหวในผู้ป่วยพาร์กินสัน
- เพื่อศึกษาผลของการรักษาการติดเชื้อเอชพีต่ออาการที่นอกเหนือการเคลื่อนไหวในผู้ป่วยพาร์กินสัน
- เพื่อศึกษาผลของการรักษาการติดเชื้อเอชพีต่อคุณภาพชีวิตของผู้ป่วยพาร์กินสัน

ประเภทของโครงการวิจัย

เป็นการวิจัยทางคลินิกประเภทตามรุ่นตามแผนแบบไม่มีกลุ่มควบคุม

อาสาสมัคร

การคำนวณขนาดตัวอย่าง

คำนวณขนาดตัวอย่างโดยอ้างอิงจากข้อมูลการศึกษาเดิมโดยใช้สูตรเพื่อหาความแตกต่างของค่าเฉลี่ยก่อนและหลังการรักษาโดยใช้สูตร โดยมีระดับความเชื่อมั่นที่ร้อยละ 80

$$n = \left[\frac{(Z_{\alpha/2} + Z_{\beta})\sigma}{\Delta} \right]^2$$

ได้จำนวนผู้ป่วยที่มีการติดเชื้อเอชพี อย่างน้อย 15 ราย เมื่ออ้างอิงจากการศึกษาเดิมพบว่ามีผู้ป่วยพาร์กินสันที่ติดเชื้อเอชพี ประมาณร้อยละ 40 ดังนั้นต้องใช้ผู้ป่วยพาร์กินสันทั้งหมดประมาณ 40 ราย

ผู้ป่วยพาร์กินสันทุกรายที่เข้ารับการรักษาที่คลินิกประสาทวิทยา โรงพยาบาลธรรมศาสตร์ จะได้รับการประเมินและคัดกรองเพื่อเข้าร่วมงานวิจัย

เกณฑ์การคัดเลือกผู้ร่วมวิจัย/อาสาสมัคร

- ผู้ป่วยพาร์กินสันที่ได้รับการวินิจฉัยตามเกณฑ์ของ UK Parkinson’s Disease Society Brain Bank Diagnostic Criteria (UKPDSBB)
- อายุมากกว่าหรือเท่ากับ 18 ปี

- มีอาการตอบสนองต่อยาที่ไม่สม่ำเสมอ
- ได้รับการรักษาด้วยยาลีโวโดปา

เกณฑ์การคัดออกผู้ร่วมวิจัย/อาสาสมัคร

- ผู้ป่วยพาร์กินโซนิซึ่มที่มีสาเหตุหรือผู้ป่วยพาร์กินสันเทียม
- ผู้ป่วยที่มีปัญหาสมองเสื่อม
- ผู้ป่วยพาร์กินสันที่มีภาวะทุพพลภาพมาก (Hoehn and Yahr scale 5)
- ผู้ป่วยที่มีปัญหาการกลืนลำบาก
- ผู้ป่วยที่มีประวัติของโรคกระเพาะอาหารหรือเคยได้รับการผ่าตัดกระเพาะอาหารมาก่อน
- ผู้ป่วยที่เคยได้รับการรักษาเชื้อเอชพีมาก่อน
- ผู้ป่วยที่มีประวัติแพ้ยากลุ่ม amoxycillin, clarithromycin และ omeprazole
- ผู้ป่วยตั้งครรภ์

กระบวนการวิจัยและกระบวนการเก็บข้อมูล

การตรวจครั้งที่	กระบวนการ
1	- แจ้งวิธีการศึกษาวิจัย ผลดี ผลเสียที่อาจจะเกิดขึ้นได้ระหว่างการทำวิจัย<br>- เซ็นใบยินยอมเข้าร่วมงานวิจัย<br>- เก็บข้อมูลพื้นฐาน<br>- ผู้ป่วยจะได้รับการสอนการทำตารางปฏิทินสำหรับผู้ป่วยพาร์กินสัน โดยผู้ป่วยจะต้องทำปฏิทินอย่างน้อย 3 วันก่อนเข้ามาเพื่อการตรวจครั้งที่ 1<br>- ผู้ป่วยที่รับประทานยาปฏิชีวนะอยู่จะต้องหยุดยาอย่างน้อย 4 สัปดาห์<br>- ผู้ป่วยที่รับประทานยาลดกรดจะต้องหยุดยาอย่างน้อย 2 สัปดาห์<br>- ทำการนัดหมายเพื่อเข้ารับการตรวจครั้งที่ 2
2	- งดน้ำงดอาหารและยาที่ใช้ในการรักษาพาร์กินสันอย่างน้อย 8 ชั่วโมงเพื่อเข้ารับการประเมินในตอนเช้า<br>- ประเมินอาการทางการเคลื่อนไหว UPDRS และ time up and go ในขณะออฟ<br>- ทดสอบการติดเชื้อเอชพี โดย ^{13}C-urea breath test (UBT) ใช้เวลาประมาณ 30 นาที

	- รับประทานยาลีโวโดปาขนาดปกติที่ผู้ป่วยรับประทานในมื้อเช้าพร้อมกับน้ำเปล่า<br>- ประเมินระยะเวลาที่ยาลีโวโดปาเริ่มออกฤทธิ์ และออกฤทธิ์สูงสุด<br>- ประเมินอาการทางการเคลื่อนไหว UPDRS และ time up and go ในขณะออน (ในขณะที่ผู้ป่วยรู้สึกว่ายาออกฤทธิ์สูงสุด หรือ 90 นาทีหลังรับประทานยา)<br>- ประเมินอาการที่นอกเหนือจากการเคลื่อนไหว<br>- ประเมินอาการทางระบบทางเดินอาหาร<br>- ประเมินอาการแวริ่งออฟ ด้วยแบบสอบถามภาษาไทย<br>- ประเมินคุณภาพชีวิตของผู้ป่วยพาร์กินสันด้วย พีดีคิว-8<br>- ผู้ป่วยที่ได้ผลบวกจากการตรวจ UBT จะได้รับการรักษาเพิ่มเติมด้วยยา amoxicillin (1 mg twice daily), clarithromycin (500 mg twice daily) และ omeprazole (40 mg twice daily) นาน 2 สัปดาห์<br>- ทำการนัดหมายเพื่อเข้ารับการประเมินที่ 6 สัปดาห์<br>- ผู้ตรวจและทำการประเมินไม่ทราบผลการตรวจเอชพีของผู้ป่วย
**3 (≥ 6 สัปดาห์)**	- นัดเพื่อเข้ารับการประเมินเช่นเดียวกับการประเมินครั้งที่ 2<br>- ทดสอบการติดเชื้อเอชพี โดย ^{13}C-urea breath test (UBT) เพื่อประเมินการรักษาหาย<br>- ประเมินผลการรักษาโดยรวม<br>- ผู้ตรวจและทำการประเมินไม่ทราบผลการตรวจเอชพีของผู้ป่วย

การวัดผล

การวัดผลของวัตถุประสงค์หลัง

- การเปลี่ยนแปลงของระยะเวลา “ออน” และ “ออฟ” ภายหลังการรักษา
- การเปลี่ยนแปลงของระยะเวลาเริ่มออกฤทธิ์และออกฤทธิ์สูงสุดของยาลีโวโดปา
- การเปลี่ยนแปลงของอาการแวริ่งออฟ

การวัดผลของวัตถุประสงค์รอง

- การเปลี่ยนแปลงของคะแนนการเคลื่อนไหว UPDRS
- การเปลี่ยนแปลงของคะแนนอาการที่นอกเหนือจากการเคลื่อนไหวและอาการในระบบทางเดินอาหาร

- การเปลี่ยนแปลงของระดับคุณภาพชีวิต

การวิเคราะห์ผลทางสถิติ

- วิเคราะห์ข้อมูลโดยใช้โปรแกรม SPSS เวอร์ชั่นที่ 17.0
- ข้อมูลเชิงพรรณนานำเสนอโดยค่าเฉลี่ย ค่าเบี่ยงเบนมาตราฐานและร้อยละ
- ข้อมูลเปรียบเทียบความแตกต่างของค่ากลางระหว่างกลุ่มที่มีเอชพีเป็นบวกและลบ ใช้สถิติ Mann-Whitney test หรือ independent t-test และ chi-square ตามความเหมาะสมของชนิดข้อมูล
- ข้อมูลเปรียบเทียบความแตกต่างของค่ากลางระหว่างก่อนและหลังการรักษาเอชพี ใช้สถิติ Wilcoxon paired rank-sum test หรือ dependent t-test ตามความเหมาะสม
- ข้อมูลการศึกษาความสัมพันธ์ใช้วิธี correlational analysis
- การทดสอบสมมติฐานใช้การทดสอบแบบสองทาง โดย $p<0.05$ ถือว่ามีนัยสำคัญทางสถิติ

ทุนวิจัย

การศึกษานี้ได้รับทุนวิจัยทั่วไปจากมหาวิทยาลัยธรรมศาสตร์และทุนสนับสนุนวิจัยบางส่วนจากชมรมพาร์กินสัน ประเทศไทย

โครงการวิจัย

**ชื่อเรื่อง**	ผลการกำจัดเชื้อเฮลิโคแบคเตอร์ ไพโลไร ต่อการตอบสนองต่อยาไม่สม่ำเสมอในผู้ป่วยพาร์กินสัน
**ผู้รับผิดชอบ**	รศ.นพ. ประวีณ โล่เลขา<br>นพ. ธนาคาร ศรีพนม<br>ศ.นพ.รัฐกร วิไลชนม์
**วัตถุประสงค์หลัก**	เพื่อศึกษาผลของการรักษาการติดเชื้อเอชพีต่อการตอบสนองต่อยาไม่สม่ำเสมอในผู้ป่วยพาร์กินสัน
**วัตถุประสงค์รอง**	เพื่อศึกษาผลของการรักษาการติดเชื้อเอชพีต่ออาการทางการเคลื่อนไหว อาการที่นอกเหนือจากการเคลื่อนไหว และคุณภาพชีวิตของผู้ป่วยพาร์กินสันที่มีการตอบสนองต่อยาไม่สม่ำเสมอ
**รูปแบบการศึกษา**	การวิจัยทางคลินิกประเภทตามรุ่นตามแผนแบบไม่มีกลุ่มควบคุม
**อาสาสมัคร**	ผู้ป่วยพาร์กินสันที่มีการตอบสนองต่อยาไม่สม่ำเสมอจำนวน 40 ราย
**การรักษา**	การกำจัดเชื้อเอชพี ด้วยยาสามชนิด ขนาดมาตรฐานเป็นเป็นเวลา 2 สัปดาห์ (ยาอะม็อกซีซิลลิน ขนาด 1 กรัม วันละ 2 ครั้ง ยาคลาริโธรมัยซิน ขนาด 500 มิลลิกรัม วันละ 2 ครั้ง และยาโอเมพราโซล ขนาด 40 มิลลิกรัม วันละ 2 ครั้ง)
**กระบวนการวิจัย**	1 เก็บข้อมูลพื้นฐาน ให้คำยินนอม และอธิบายกระบวนการวิจัย<br>2 ประเมินอาการครั้งที่ 1<br>3 ประเมินอาการครั้งที่ 2 ห่างจากครั้งแรกอย่างน้อย 6 สัปดาห์
**การวัดผล**	การประเมิน ^{13}C-urea breath test (UBT)<br>ระยะเวลา “ออน” และ “ออฟ”<br>ระยะเวลาเริ่มออกฤทธิ์และออกฤทธิ์สูงสุดของยาลีโวโดปา<br>แบบสอบถามแวริ่งออฟภาษาไทย<br>ประเมินอาการทางการเคลื่อนไหว<br>ประเมินการเดินและเวลาที่ใช้ในการเดิน<br>ประเมินอาการที่นอกเหนือจากการเคลื่อนไหว<br>ประเมินอาการทางระบบทางเดินอาหาร<br>ประเมินระดับคุณภาพชีวิตของผู้ป่วย

วันที่..

แบบเก็บข้อมูลผู้ป่วยโรคพาร์กินสัน รพ. ธรรมศาสตร์เฉลิมพระเกียรติ

1-1 ข้อมูลทั่วไป

1.1 ....

1.2 อายุ..........................ปี 1.3 เพศ ☐ ชาย ☐ หญิง

1.4 ค่ารักษาพยาบาลเบิกได้หรือไม่ ☐ ราชการ ☐บัตรทอง ☐ ประกันสังคม ☐ จ่ายเอง

1.5. ประวัติโรคพาร์กินสันในครอบครัว ☐ ไม่มี ☐ มี...

1.6 ระดับการศึกษา ....

1-2. Schwab & England ADL scale

Completely independent	☐ 100% Normal<br>☐ 90% Some slowness<br>☐ 80 % Takes twice as long, conscious of difficulty and slow<br>☐ 70% 3-4 times as long in some
Some dependency	☐ 60 % Can do most chores, but slow and much effort. Error
More dependent	☐ 50% Slower, difficulty in everything
Very dependent	☐ 40 % Can assist with all chores but few alone<br>☐ 30 % Dose a few chores alone or begins alone, need help<br>☐ 20 % Nothing alone
Totally dependent	☐ 10 %
Vegetative, Bedridden	☐ 0 %

1-3. **ข้อมูลการรักษาโรคพาร์กินสัน**

อายุที่เริ่มมีอาการของโรคพาร์กินสัน...ปี

ระยะเวลาที่มีอาการของโรคพาร์กินสัน...ปี

อาการของโรคพาร์กินสัน ☐ tremor dominant ☐ PIGD ☐ Unidentified

ขอขอบคุณที่ท่านช่วยตอบแบบสอบถามจนเสร็จสมบูรณ์

วันที่...

ยาที่รับประทาน	ขนาดที่รับประทานต่อวัน	Conversion factor	Levodopa equivalent dose
Levodopa (IR)		x 1	
Levodopa (HBS)		x 0.75	
Entacapone/ Stalevo		LD x 0.33	
Pramipexole		x 100	
Ropinirole		x 20	
Rotigotine		x 30	
Peribidil		x 1	
Bromocriptine		x 10	
Rasagiline		x 100	
Apomorphine		x 10	
Others			

Antidementia ☐ Donepezil ☐ Rivastigmine ☐ Galantamine ☐ Memantine

Antidepressant ☐ ……………………………………………………………………………..

Antipsychotic ☐ Haloperidol ☐ Risperdal ☐ Quetiapine ☐ Others………………………………………

Anxiolytics ☐ Clonazepam ☐ Lorazepam ☐ Alprazolam ☐ Sleep medication

Others ☐ Anticholinergics

Anti-Acid ☐ ……………………………… หยุด2สัปดาห์

Anti-biotics ☐ ……………………………… หยุด 4 สัปดาห์

1-4. Motor complication

4.1 Predictable wearing off ☐ Yes ☐ No EOD score …………………………………………..

4.2 Unpredictable On/Off ☐ Yes ☐ No

ขอขอบคุณที่ท่านช่วยตอบแบบสอบถามจนเสร็จสมบูรณ์

วันที่..

4.3 Dose failure ☐ Yes ☐ No

4.4 Peak dose dyskinesia ☐ Yes ☐ No

4.5 Biphasic dyskinesia ☐ Yes ☐ No

4.6 Off dystonia ☐ Yes ☐ No

4.7 Morning akinesia ☐ Yes ☐ No

1-5 PDQ8, 1-6 GI complain score, 1-7 NMSQ แยกเก็บ

ครั้งที่ 2 ทดสอบ UBT

3-1. PD diary ครั้งที่ 1 On…………% Off…………% Dyskinesia ……….%

3-2. ผลการทดสอบ UBT ☐ Positive ☐ Negative

3-3. Off UPDRS motor score ...…………………………….

3-4. Onset time of levodopa action ……………………………….. นาที

3-5. On UPDRS motor score ……………………………….

3-6. Time up and go test ……………………………… นาที

ให้การรักษาในกลุ่ม UBT +ve

4-1. PD diary ครั้งที่ 2 On…………% Off…………% Dyskinesia ……….%

4.2 Side effect of therapy ☐ Yes ………………………………. ☐ No

4-3. Off UPDRS motor score ...…………………………….

4-4. Onset time of levodopa action ……………………………….. นาที

4-5. On UPDRS motor score ……………………………….

ขอขอบคุณที่ท่านช่วยตอบแบบสอบถามจนเสร็จสมบูรณ์

วันที่...

4-6. Time up and go test ……………………………… นาที

Motor complication

4.1 Predictable wearing off ☐ Yes ☐ No EOD score …………………………………………..

4.2 Unpredictable On/Off ☐ Yes ☐ No

4.3 Dose failure ☐ Yes ☐ No

4.4 Peak dose dyskinesia ☐ Yes ☐ No

4.5 Biphasic dyskinesia ☐ Yes ☐ No

4.6 Off dystonia ☐ Yes ☐ No

4.7 Morning akinesia ☐ Yes ☐ No

1-5 PDQ8, 1-6 GI complain score, 1-7 NMSQ แยกเก็บ

4-8. ผลการทดสอบ UBT-post ☐ Positive ☐ Negative

ขอขอบคุณที่ท่านช่วยตอบแบบสอบถามจนเสร็จสมบูรณ์

วันที่..

แบบสอบถามอาการทางระบบทางเดินอาหาร

ในช่วง 1 สัปดาห์ที่ผ่านมาท่านมีอาการเหล่านี้หรือไม่เพียงใด

1. อาการแสบร้อนกลางอก	ไม่มีอาการ มีอาการปานกลาง มีอาการมาก<br>0 1 2 3 4 5 6 7 8 9 10
2. อาการท้องอืดท้องเฟ้อ	0 1 2 3 4 5 6 7 8 9 10
3. อาการคลื่นไส้	0 1 2 3 4 5 6 7 8 9 10
4. อาการอาเจียน	0 1 2 3 4 5 6 7 8 9 10
5. อาการปวดท้อง	0 1 2 3 4 5 6 7 8 9 10
6. อาการท้องเสีย	0 1 2 3 4 5 6 7 8 9 10
7. อาการท้องผูก	0 1 2 3 4 5 6 7 8 9 10

ขอขอบคุณที่ท่านช่วยตอบแบบสอบถามจนเสร็จสมบูรณ์

แบบประเมินอาการช่วงยาหมดฤทธิ์ “แวริ่ง ออฟ” หรืออาการต่างๆที่กำเริบของโรคพาร์กินสัน ก่อนที่จะรับประทานยามื้อต่อไป “เอน ออฟ โดส”

Thai version of the 9-item Wearing-off Questionnaire (TWOQ-9)

End-of-dose Questionnaire (EOD)

แบบประเมินที่ใช้คัดกรองอาการยาหมดฤทธิ์ (wearing - off) ทั้งอาการที่เกี่ยวข้องกับการเคลื่อนไหว (motor symptoms) และอาการที่ไม่เกี่ยวข้องกับการเคลื่อนไหว (non - motor symptoms) โดยให้เลือกอาการที่เกิดขึ้น ในช่วงที่ยาหมดฤทธิ์ และอาการนั้นๆ จะต้องดีขึ้นเมื่อยาออกฤทธิ์ จึงจะเข้าเกณฑ์การวินิจฉัย wearing-off จากการศึกษาแบบ multicenter, cross-sectional พบว่า WOQ-9 มี sensitivity 96.2% และ specificity 40.9%

กรุณาลงเครื่องหมาย ✓ ในช่อง เพื่อเลือกอาการช่วงที่ยาหมดฤทธิ์ “แวริ่ง ออฟ” ใดๆ ที่เกิดขึ้นในระหว่างวันของท่าน ถ้าท่านมีอาการช่วงยาหมดฤทธิ์ “แวริ่ง ออฟ” มากกว่า 2 อาการ ท่านสามารถเลือกได้ตามอาการที่เกิดขึ้น และลงเครื่องหมาย ✓ ในช่อง อีกครั้ง หากอาการดังกล่าวดีขึ้นหลังรับประทานยามื้อถัดไป

อาการช่วงที่ยาหมดฤทธิ์ “แวร์ริ่ง ออฟ”	มีอาการ	อาการดีขึ้นหลังรับประทานยา
สั่น		
กระวนกระวาย		
อารมณ์เปลี่ยนแปลง		
เคลื่อนไหวช้า		
ใช้มือไม่คล่อง		
เกร็งทั่วไป		
คิดอะไรไม่ออก		
กล้ามเนื้อเกร็ง		
ปวดเมื่อย		

- ดัดแปลงจาก Thai Parkinson’s disease and movement disorder

แบบบันทึกอาการผู้ป่วยพาร์กินสัน โรงพยาบาลธรรมศาสตร์เฉลิมพระเกียรติ

ชื่อ-นามสกุล .... วันที่ ....

	**เวลา**	รับประทานยาลีโวโดปา 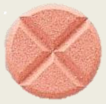	**อาการ ออฟ (ไม่ดี)** ลำตัวแข็ง เกร็ง เคลื่อนไหวลำบาก หรือสั่นมาก	**อาการ ออน (ดี)** เคลื่อนไหวได้เป็นปรกติ ใกล้เคียงปรกติ สบายตัว	**อาการยุกยิก** เคลื่อนไหวได้ แต่มีอาการส่าย/โยกไปมาที่ลำตัว ใบหน้า	**นอนหลับ**
1	6.00 - 6.29					
2	6.30 - 6.59					
3	7.00 - 7.29					
4	7.30 - 7.59					
5	8.00 - 8.29					
6	8.30 - 8.59					
7	9.00 - 9.29					
8	9.30 - 9.59					
9	10.00-10.29					
10	10.30-10.59					
11	11.00-11.29					
12	11.30-11.59					
13	12.00-12.29					
14	12.30-12.59					
15	13.00-13.29					
16	13.30-13.59					
17	14.00-14.29					
18	14.30-14.59					
19	15.00-15.29					
20	15.30-15.59					
21	16.00-16.29					
22	16.30-16.59					
23	17.00-17.29					
24	17.30-17.59					
25	18.00-18.29					
26	18.30-18.59					
27	19.00-19.29					
28	19.30-19.59					
29	20.00-20.29					
30	20.30-20.59					
31	21.00-21.29					
32	21.30-21.59					
33	22.00-22.29					
34	22.30-22.59					
35	23.00-23.29					

สำหรับแพทย์

แบบเก็บข้อมูล UPDRS III. Motor Exam รพ. ธรรมศาสตร์เฉลิมพระเกียรติ

……………………………………………..

Date/ time……………………………………

DOB & Age …………………………………

Last Meds taken ……………………………

☐ Wearing off / off symptom

Dyskinesias

☐ Upper ☐ Lower ☐ Generalized

Speech

0 = Normal.

1 = Slight loss of expression, diction and/or volume.

2 = Monotone, slurred but understandable; moderately impaired.

3 = Marked impairment, difficult to understand.

4 = Unintelligible.

Rigidity

H	
R	L

0 = Absent.

1 = Slight or detectable only when activated by mirror or other movements.

2 = Mild to moderate.

3 = Marked, but full range of motion easily achieved.

4 = Severe, range of motion achieved with difficulty.

Facial Expression

0 = Normal.

1 = Minimal hypomimia, could be "Poker Face".

2 = Slight but definitely abnormal diminution of facial expression.

3 = Moderate hypomimia; lips parted some time.

4 = Masked or fixed facies with severe or complete loss of facial expression; lips parted 1/4 inch or more.

Finger Taps

R	L

0 = Normal.

1 = Mild slowing and/or reduction in amplitude.

2 = Moderately impaired. Definite and early fatiguing. May have occasional arrests in movement.

3 = Severely impaired. Frequent hesitation in initiating movements or arrests in ongoing movement.

4 = Can barely perform the task.

Tremor at rest

H	
R	L

0 = Absent.

1 = Slight and infrequently present.

2 = Mild in amplitude and persistent.

Or moderate in amplitude, but only intermittently present.

3 = Moderate in amplitude and present most of the time.

4 = Marked in amplitude and present most of the time.

Hand Movements

R	L

0 = Normal.

1 = Mild slowing and/or reduction in amplitude.

2 = Moderately impaired. Definite and early fatiguing. May have occasional arrests in movement.

3 = Severely impaired. Frequent hesitation in initiating movements or arrests in ongoing movement.

4 = Can barely perform the task.

Action/Postural Tremor of hands

R	L

0 = Absent.

1 = Slight; present with action.

2 = Moderate in amplitude, present with action.

3 = Moderate in amplitude with posture as well as action.

4 = Marked in amplitude; interferes with feeding.

Rapid Alternating Movements of Hands

R	L

0 = Normal.

1 = Mild slowing and/or reduction in amplitude.

2 = Moderately impaired. Definite and early fatiguing. May have occasional arrests in movement.

3 = Severely impaired. Frequent hesitation in initiating movements or arrests in ongoing movement.

4 = Can barely perform the task.

Leg Agility | R | L |

0 = Normal.
1 = Mild slowing and/or reduction in amplitude.
2 = Moderately impaired. Definite and early fatiguing. May have occasional arrests in movement.
3 = Severely impaired. Frequent hesitation in initiating movements or arrests in ongoing movement.
4 = Can barely perform the task.

Postural Stability

0 = Normal.
1 = Retropulsion, but recovers unaided.
2 = Absence of postural response; would fall if not caught by examiner.
3 = Very unstable, tends to lose balance spontaneously.
4 = Unable to stand without assistance.

Arising from Chair

0 = Normal.
1 = Slow; or may need more than one attempt.
2 = Pushes self up from arms of seat.
3 = Tends to fall back and may have to try more than one time, but can get up without help.
4 = Unable to arise without help.

Body Bradykinesia and Hypokinesia

0 = None.
1 = Minimal slowness, giving movement a deliberate character; could be normal for some persons. Possibly reduced amplitude.
2 = Mild degree of slowness and poverty of movement which is definitely abnormal. Alternatively, some reduced amplitude.
3 = Moderate slowness, poverty or small amplitude of movement.
4 = Marked slowness, poverty or small amplitude of movement

Posture

0 = Normal erect.
1 = Not quite erect, slightly stooped posture; could be normal for older person.
2 = Moderately stooped posture, definitely abnormal; can be slightly leaning to one side.
3 = Severely stooped posture with kyphosis; can be moderately leaning to one side.
4 = Marked flexion with extreme abnormality of posture.

Total score

UPDRS III [ ] **/ 108**

Right limbs [ ] **/ 36**

Left limbs [ ] **/ 36**

Gait

0 = Normal.
1 = Walks slowly, may shuffle with short steps, but no festination (hastening steps) or propulsion.
2 = Walks with difficulty, but requires little or no assistance; may have some festination, short steps, or propulsion.
3 = Severe disturbance of gait, requiring assistance.
4 = Cannot walk at all, even with assistance.

Modified Hoehn and Yahr Staging:

0 = No signs of disease
1= Unilateral disease
1.5 = Unilateral plus axial involvement
2= Bilateral disease, no impairment of balance (0/4)
2.5 = Bilateral disease, mild, recovery on pull test (1/4)
3 = Bilateral disease with some postural instability, physically independent (2/4)
4 = Severe disability, still able to walk or stand unassisted (3/4)
5 = Wheelchair bound or bed ridden unless unaided

แบบสอบถามคุณภาพชีวิต สำหรับโรคพาร์กินสัน (PDQ-8)

จากการป่วยเป็นโรคพาร์กินสัน
บ่อยแค่ไหนในช่วงหนึ่งเดือนที่ผ่านมาที่คุณ...

*กรุณา**กาเครื่องหมายเพียงหนึ่งช่อง** สำหรับแต่ละคำถาม*

		ไม่เคย	เป็นบางโอกาส	เป็นบางเวลา	บ่อยๆ	เสมอๆ หรือ ไม่สามารถทำได้เลย
1.	มีความยากลำบากในการไปไหนมาไหนในที่สาธารณะ?	☐	☐	☐	☐	☐
2.	มีความยากลำบากในการแต่งตัว?	☐	☐	☐	☐	☐
3.	รู้สึกเศร้า?	☐	☐	☐	☐	☐
4.	มีปัญหากับผู้มีความสัมพันธ์ใกล้ชิดกับคุณ?	☐	☐	☐	☐	☐
5.	มีปัญหาเกี่ยวกับสมาธิของคุณ เช่น เวลาอ่านหนังสือหรือดูโทรทัศน์?	☐	☐	☐	☐	☐
6.	รู้สึกไม่สามารถสื่อสารกับผู้คนได้อย่างเหมาะสม?	☐	☐	☐	☐	☐
7.	มีอาการเจ็บปวดจากกล้ามเนื้อเป็นตะคริวหรือเกร็งตัว?	☐	☐	☐	☐	☐
8.	รู้สึกอับอายในที่สาธารณะเนื่องจากเป็นโรคพาร์กินสัน?	☐	☐	☐	☐	☐

กรุณาตรวจทานให้แน่ใจว่าคุณได้***เลือกเพียงคำตอบเดียวสำหรับแต่ละข้อ***.

ขอขอบคุณที่สละเวลาตอบแบบสอบถาม.

Final Thai (Thailand) PDQ-8

PDQ-8 © Copyright, Oxford University Innovation Limited 1998. All Rights Reserved.
The authors, being Professor Crispin Jenkinson, Professor Ray Fitzpatrick and Ms Viv Peto, have asserted their moral rights.

<u>เอกสารแสดงความยินยอมเข้าร่วมโครงการวิจัย</u>

ข้าพเจ้าขอยืนยันว่า ผู้เข้าร่วมโครงการวิจัยได้รับโอกาสในการซักถามและทุกข้อสงสัยได้รับการอธิบายอย่างถูกต้องชัดเจนแล้ว ผู้เข้าร่วมโครงการวิจัยยินยอมเข้าร่วมโครงการวิจัยด้วยความสมัครใจ

____

ชื่อตัวบรรจงผู้ยินยอมเข้าร่วมโครงการวิจัย

____

ลายเซ็นผู้ยินยอมเข้าร่วมโครงการวิจัย

วันที่ _________________________

____

ชื่อตัวบรรจงผู้ขอความยินยอมจากผู้เข้าร่วมโครงการวิจัย

____

ลายเซ็นผู้ขอความยินยอมจากผู้เข้าร่วมโครงการวิจัย

วันที่ _________________________
