# Supplementary material for: Helicobacter pylori eradication improves motor fluctuations in advanced Parkinson’s disease patients: A prospective cohort study (HP-PD trial)
Source: PLoS One. 2021 May 4;16(5):e0251042. doi: 10.1371/journal.pone.0251042 (PMC8096108; doi:10.1371/journal.pone.0251042)
Supplement: S2 Protocol — (PDF) [file pone.0251042.s004.pdf]

Study Protocol

(English translation of the Thai study protocol; for the complete study protocol see Thai version)

Effects of Helicobacter Pylori Eradication on Motor Fluctuations in patients with Parkinson's Disease

Responsible:

Associate Professor Praween Lolekha, M.D., MSc.
Thanakarn Sriphanom, M.D.
Professor Ratha-korn Vilaichone, M.D., Ph.D.

Neurology Division, Department of Medicine
Faculty of Medicine, Thammasat University
95 Moo 8 Paholyotin Road, Amphur Klongluang,
Pathumthani, 12120, Thailand.

Background

Parkinson's disease (PD) is one of the most common neurodegenerative disorders that commonly affect elderly people. According to the previous study, the prevalence of PD in Thailand was approximately 424.57 cases per 100,000 populations. Despite enormous advanced knowledge of PD, L-dopa (levodopa) remains the most effective treatment for PD. Almost all PD patients require varying doses of L-dopa to manage their motor symptoms and maintain an acceptable quality of life. However, chronic L-dopa use in PD is associated with motor complications, including motor fluctuations and L-dopa-induced dyskinesia. After 5 years of L-dopa exposure, more than half of PD patients may experience some degree of these problems that significantly affects their functionality and quality of life. Although PD mainly affects the motor system, it has been well established that PD is also associated with several non-motor symptoms (NMS). Gastrointestinal (GI) dysfunction is one of the most problematic NMS in PD and may precede the appearance of motor symptoms by several years. Recent evidence suggested an integral role of the microbiota-gut-brain axis for the early pathogenesis and progression of PD. Also, several studies have shown an increased risk of PD in populations with chronic constipation and inflammatory bowel disease.

Helicobacter pylori (HP) is a Gram-negative bacterium that characteristically causes sustained inflammation of the gastric mucosa, which is associated with many GI diseases such as peptic ulcer, gastroduodenitis and fatal GI diseases especially gastric cancer. The prevalence of HP infection in the general Thai population was approximately 45.9%. Men showed higher risk for HP infection than women. The infection rate was approximately 40-50% in all age groups with the highest prevalence of 49.3% for patients aged 40-49 years. A recent population-based study has shown an increased risk of PD in chronic HP infection. Also, meta-analysis has shown a significantly higher prevalence of HP infection in PD patients; furthermore, HP-infected PD patients demonstrated poorer control of motor symptoms than non-infected patients. As GI dysfunction and L-dopa bioavailability play an important role in the pathophysiological changes underlying motor fluctuations, the eradication of HP might positively influence clinical outcome in the treatment of PD.

Objective

Primary objective

- To evaluate the therapeutic effects of HP eradication on motor fluctuations in advanced PD patients

Secondary objectives

- To evaluate the therapeutic effects of HP eradication on motor symptoms
- To evaluate the therapeutic effects of HP eradication on non-motor symptoms
- To evaluate the therapeutic effects of HP eradication on quality of life

Study design

A prospective cohort, single-arm, open-label clinical study.

Participants

Sample size calculation

The estimated sample size was determined according to the previous studies with a level of significance of 5% and the statistical power of 80%. The target sample size was set at 40 PD patients, in order to get at least 15 patients with HP infection.

Consecutive PD patients were recruited and screened by a movement disorder specialist at the movement disorder clinic, Thammasat University Hospital

Inclusion criteria

- Diagnosed with idiopathic PD by the UK Parkinson's Disease Society Brain Bank (UKPDSBB) criteria
- Age ≥ 18 years
- Current presence of motor fluctuations
- Receiving treatment with L-dopa

Exclusion criteria

- Atypical parkinsonism or secondary parkinsonism
- Severe cognitive dysfunction or dementia
- Severe disability (Hoehn and Yahr scale 5)
- Severe dysphagia
- History of gastric/duodenal lesions or prior gastric surgery
- Prior HP eradication therapy
- Allergic to amoxicillin, clarithromycin or omeprazole
- Pregnancy

Method

Data collection process

Visit	Process
1	- Inform consent<br>- Patients who eligible to the study were advised to do the PD diary, which is to record their daily on and off time for at least 3 days before the assessment day 1.<br>- Discontinue antibiotics (if any) for 4 weeks<br>- Discontinue proton-pump inhibitor (if any) for 2 weeks<br>- Appointment for the assessment date.
2	- Discontinue all anti-PD medications and fasting overnight or at least 8 hours before the morning assessment.<br>- Assess UPDRS 'off' motor score, time up and go<br>- ^{13}C-urea breath test (UBT)<br>- Take a usual morning dose of L-dopa with still water<br>- Evaluate L-dopa 'onset' time and 'peak' time<br>- Assess UPDRS 'on' motor score, time up and go (during the peak time or at 90 min after oral L-dopa intake, whichever comes earlier.)<br>- Assess Non-motor symptoms (NMS application)<br>- Assess GI symptoms score (visual analog scale)<br>- Assess Thai wearing-off question (Thai version of the 9-item Wearing-off Questionnaire: TWOQ-9)

	- Assess PD quality of life (PDQ-8: license 868976)<br>- Patients with positive UBT were additionally received standard triple therapy with amoxicillin (1 mg twice daily), clarithromycin (500 mg twice daily) and omeprazole (40 mg twice daily) for 2 weeks.<br>- Appointment for the reassessment date (≥ 6 weeks)<br>- The outcome evaluators were blind to the result of HP infection status.
3 (≥6 weeks)	- Reassessment with the same protocol as day 1<br>- Reevaluate ^{13}C-urea breath test (UBT) for HP eradication<br>- Assess the clinical global impression scale (CGI)<br>- The outcome evaluators were blind to the result of HP infection status.

Outcome measurement

Primary outcome

- Change of daily "on" and "off" time from baseline
- Change of L-dopa "onset" and "peak" time from baseline
- Change of wearing-off symptoms

Secondary outcome

- Change of UPDRS "on" and "off" motor score
- Change of non-motor symptom and GI symptoms score
- Change of PDQ-8 score

Statistical analysis

- Statistical analysis was performed using SPSS version 17.0 (SPSS Inc., Chicago, IL, USA).
- Descriptive statistical values were presented as mean standard deviation (SD) and percentage values.
- The independent t-test, Mann-Whitney test or chi-squared test was used to assess differences in various parameters between HP positive and HP negative groups.

- The dependent sample t-test or Wilcoxon matched-pairs signed rank test was used to assess the different outcomes between pre- and post-HP eradication in the HP positive group.
- Correlation analysis and multiple linear regression analysis were used to assess the associated factors of HP infection.
- All tests were two-sided and a probability value of $p < 0.05$ was statistically significant.

Funding

This study was supported by a grant from Thammasat University and Thai Parkinson’s Disease and Movement Disorder Society (Thai-PDMDS).

STUDY OUTLINE

**Title**	Effects of Helicobacter Pylori Eradication on<br>Motor Fluctuations in patients with<br>Parkinson's Disease
**Responsible**	Assoc. Prof. Dr. Praween Lolekha<br>Dr. Thanakarn Sriphanom<br>Professor Dr. Ratha-korn Vilaichone
**Primary objective**	To evaluate the therapeutic effects of HP eradication on motor fluctuations in advanced PD patients
**Secondary objectives**	To evaluate the therapeutic effects oh HP eradication on motor, non-motor symptoms, and quality of life of advanced PD patients
**Design**	A prospective cohort, single-arm, open-label clinical study.
**Subjects**	40 individuals with idiopathic Parkinson's disease with motor fluctuations
**Intervention**	2-week regiment of standard triple therapy with amoxicillin (1 mg twice daily), clarithromycin (500 mg twice daily) and omeprazole (40 mg twice daily)
**Assessments**	1 Baseline<br>2 Assessment day 1<br>3 Assessment day 2; 6 weeks follow-up
**Outcome measures**	^{13}C-urea breath test (UBT)<br>Daily 'on' and 'off' time<br>L-dopa "onset" and "peak" time<br>Thai wearing-off questionnaire<br>UPDRS motor score<br>Time up and go<br>Non-motor symptoms score application<br>Gastrointestinal symptoms score<br>PDQ-8
