# Supplementary material for: Helicobacter pylori eradication improves motor fluctuations in advanced Parkinson’s disease patients: A prospective cohort study (HP-PD trial)
Source: PLoS One. 2021 May 4;16(5):e0251042. doi: 10.1371/journal.pone.0251042 (PMC8096108; doi:10.1371/journal.pone.0251042)
Supplement: Attachment — Submitted filename: Response to Reviewers.docx [file pone.0251042.s005.docx]

Dear Editor,

I am pleased to resubmit the revised version PONE-D-20-33347; “Helicobacter pylori eradication improves motor fluctuations in advanced Parkinson's disease patients: A prospective cohort study (HP-PD trial)”, for publication.

I appreciated the constructive feedback of the editor and reviewers. I have addressed each of their concerns as outlined below.

**Journal Requirements:**

When submitting your revision, we need you to address these additional requirements.

1. Please ensure that your manuscript meets PLOS ONE's style requirements, including those for file naming. The PLOS ONE style templates can be found at

https://journals.plos.org/plosone/s/file?id=wjVg/PLOSOne_formatting_sample_main_body.pdf and

https://journals.plos.org/plosone/s/file?id=ba62/PLOSOne_formatting_sample_title_authors_affiliations.pdf

**Response:** Thank you for your suggestion. We have corrected the manuscript to meets PLOS ONE’s style requirements, including file naming and affiliations formatting.

2. Please provide additional details regarding participant consent. In the ethics statement in the Methods and online submission information, please ensure that you have specified what type you obtained (for instance, written or verbal, and if verbal, how it was documented and witnessed). If your study included minors, state whether you obtained consent from parents or guardians. If the need for consent was waived by the ethics committee, please include this information.
Once you have amended this/these statement(s) in the Methods section of the manuscript, please add the same text to the “Ethics Statement” field of the submission form (via “Edit Submission”).

For additional information about PLOS ONE ethical requirements for human subjects’ research, please refer to http://journals.plos.org/plosone/s/submission-guidelines#loc-human-subjects-research.

**Response:** We appreciate your comment. We have added this information in the methods section of the manuscript and ethics statement in the online submission information as the following paragraph.

“All participants were verbally explained regarding the study before signing the consent form. Written informed consent was obtained from all subjects prior to enrolment.”

3. Thank you for stating the following in the Acknowledgments Section of your manuscript:

"We thank the Digestive Diseases Research Centre (DRC) at Thammasat University
Hospital and also Thai Otsuka Pharmaceutical Co., Ltd, who provided the infrared
spectrophotometer for the study."

We note that you have provided funding information that is not currently declared in your Funding Statement. However, funding information should not appear in the Acknowledgments section or other areas of your manuscript. We will only publish funding information present in the Funding Statement section of the online submission form.

Please remove any funding-related text from the manuscript and let us know how you would like to update your Funding Statement. Currently, your Funding Statement reads as follows:

"This study was supported by a grant from
Thammasat University Research Fund (TU Research Scholar, Contract No. 2/4/2562) and Thai Parkinson’s Disease and Movement Disorder Society (Thai-PDMDS). "

Additionally, because some of your funding information pertains to [commercial funding//patents], we ask you to provide an updated Competing Interests statement, declaring all sources of commercial funding.

In your Competing Interests statement, please confirm that your commercial funding does not alter your adherence to PLOS ONE Editorial policies and criteria by including the following statement: "This does not alter our adherence to PLOS ONE policies on sharing data and materials.” as detailed online in our guide for authors  [http://journals.plos.org/plosone/s/competing-interests](about:blank).  If this statement is not true and your adherence to PLOS policies on sharing data and materials is altered, please explain how.

Please include the updated Competing Interests Statement and Funding Statement in your cover letter. We will change the online submission form on your behalf.

Please know it is PLOS ONE policy for corresponding authors to declare, on behalf of all authors, all potential competing interests for the purposes of transparency. PLOS defines a competing interest as anything that interferes with, or could reasonably be perceived as interfering with, the full and objective presentation, peer review, editorial decision-making, or publication of research or non-research articles submitted to one of the journals. Competing interests can be financial or non-financial, professional, or personal. Competing interests can arise in relationship to an organization or another person. Please follow this link to our website for more details on competing interests: [http://journals.plos.org/plosone/s/competing-interests](about:blank)

**Response:** Thank you for your suggestion. We have updated and removed funding-related text in the acknowledgment, funding statement, and competing interests as follows:

**Acknowledgements**

We thank the Digestive Diseases Research Centre (DRC) at Thammasat University Hospital for their assistance with the UBT tests. We would also like to show our gratitude to the patients and their families who took part and participated in the study.

**Funding Statement**

This study was supported by a grant from Thammasat University Research Fund (TU Research Scholar, Contract No. 2/4/2562) and Thai Parkinson’s Disease and Movement Disorder Society (Thai-PDMDS). The funders had no role in study design, data collection and analysis, decision to publish, or preparation of the manuscript.

**Competing interests**

The 13^C^-urea breath test and infrared spectrophotometer used in the study was provided by Thai Otsuka Pharmaceutical Co., Ltd. This does not alter our adherence to PLOS ONE policies on sharing data and materials.

4. Please include captions for your Supporting Information files at the end of your manuscript, and update any in-text citations to match accordingly. Please see our Supporting Information guidelines for more information: [http://journals.plos.org/plosone/s/supporting-information](about:blank).

**Response:** Thank you for your suggestion. We have updated captions for supporting information files at the end of the manuscript.

**Reviewers' comments:**

Reviewer's Responses to Questions

**Comments to the Author**

1. Is the manuscript technically sound, and do the data support the conclusions?

The manuscript must describe a technically sound piece of scientific research with data that supports the conclusions. Experiments must have been conducted rigorously, with appropriate controls, replication, and sample sizes. The conclusions must be drawn appropriately based on the data presented.

Reviewer #1: Yes

Reviewer #2: Yes

2. Has the statistical analysis been performed appropriately and rigorously?

Reviewer #1: Yes

Reviewer #2: Yes

3. Have the authors made all data underlying the findings in their manuscript fully available?

The [PLOS Data policy](http://www.plosone.org/static/policies.action#sharing) requires authors to make all data underlying the findings described in their manuscript fully available without restriction, with rare exception (please refer to the Data Availability Statement in the manuscript PDF file). The data should be provided as part of the manuscript or its supporting information or deposited to a public repository. For example, in addition to summary statistics, the data points behind means, medians and variance measures should be available. If there are restrictions on publicly sharing data—e.g. participant privacy or use of data from a third party—those must be specified.

Reviewer #1: No

Reviewer #2: Yes

**Response:** Thank you for your suggestion. We have updated tables and data underlying the findings in the manuscript as suggestion.

4. Is the manuscript presented in an intelligible fashion and written in standard English?

PLOS ONE does not copyedit accepted manuscripts, so the language in submitted articles must be clear, correct, and unambiguous. Any typographical or grammatical errors should be corrected at revision, so please note any specific errors here.

Reviewer #1: Yes

Reviewer #2: Yes

5. Review Comments to the Author

Please use the space provided to explain your answers to the questions above. You may also include additional comments for the author, including concerns about dual publication, research ethics, or publication ethics. (Please upload your review as an attachment if it exceeds 20,000 characters)

**Reviewer #1:** This is an interesting and important study. My comments are as follows:

**1. The authors reported the changes of UPDRS motor scores and GI symptoms scores after H. pylori eradication (before and after). It is suggested that the authors may add a table to compare patients receiving eradication therapy versus those not receiving eradication therapy (HP negative group).**

**Response:** We appreciate your important suggestion. We have compared general demographic data of the study population between HP positive (all have received eradication therapy) and HP negative group in Table 1. Also, we compared the successful HP eradication group and HP negative group's clinical symptoms in Table 4 as your suggestion.

**Table 4. Clinical symptoms between patients with *Helicobacter pylori* (HP) negative and HP positive achieved the HP eradication.**

|  | **HP negative**  **(*n*=18)** | **HP eradication**  **(*n*=17)** | ***p*** |
| --- | --- | --- | --- |
| Total TWOQ-9 score | 4.7 ± 2.0 | 4.4 ± 1.7 | 0.625 |
| Total GI symptom score | 15.4 ± 8.1 | 8.1 ± 4.5 | **0.003** |
| - Heartburn | 2.4 ± 2.8 | 0.6 ± 1.4 | **0.024** |
| - Bloating | 3.3 ± 2.7 | 1.1 ± 1.6 | **0.007** |
| - Nausea | 0.6 ± 1.5 | 0.2 ± 0.5 | 0.267 |
| - Vomiting | 0.1 ± 0.5 | 0.1 ± 0.5 | 0.968 |
| - Abdominal pain | 1.6 ± 2.5 | 0.9 ± 2.2 | 0.404 |
| - Diarrhea | 0.5 ± 1.2 | 0.0 ± 0.0 | 0.096 |
| - Constipation | 6.9 ± 3.5 | 5.2 ± 2.8 | 0.115 |
| Total NMSQ score | 37.9 ± 20.9 | 29.3 ± 14.1 | 0.173 |
| - Dysphagia | 1.3 ± 1.3 | 0.3 ± 0.6 | **0.008** |
| PDQ-8 score | 9.2 ± 6.8 | 8.6 ± 5.2 | 0.759 |
| ‘Off’ UPDRS motor score | 29.8 ± 14.8 | 26.0 ± 10.0 | 0.388 |
| ‘On’ UPDRS motor score | 15.1 ± 11.3 | 10.6 ± 5.0 | 0.146 |
| Time up and go (sec) | 12.1 ± 6.3 | 9.8 ±2.8 | 0.289 |
| L-Dopa onset time (min) | 15.4 ± 10.0 | 19.5 ± 9.6 | 0.229 |
| L-Dopa peak time (min) | 34.5 ± 10.5 | 34.9 ± 13.8 | 0.979 |
| Daily ‘on’ time (h) | 12.0 ± 2.4 | 11.8 ± 2.0 | 0.781 |
| Daily ‘off’ time (h) | 3.9 ± 2.2 | 4.0 ± 2.1 | 0.819 |
| Dyskinesia (h) | 1.5 ± 2.3 | 1.3 ± 1.8 | 0.802 |

TWOQ-9: Thai version of the 9-item Wearing-off Questionnaire; GI: gastrointestinal; NMSQ: Non-Motor Symptoms Questionnaire; PDQ: Parkinson’s Disease Questionnaire; UPDRS: Unified Parkinson's Disease Rating Scale; L-Dopa: levodopa

**2. The case number of patients receiving eradication therapy is small (N=17), whereas the authors conducted multiple comparisons in the statistical analysis. Multiple testing correction might be needed or at least should be addressed in the discussion.**

**Response:** We appreciate your important comment, and we are concerned about this issue. We have performed multiple testing correction by Bonferroni adjustment in our statistic data and revealed the statistically significant difference in total TWOQ-9 score before and after eradication therapy. We have mentioned this issue in the results section and also addressed this limitation in the discussion as follows:

**Results**

**“**Multiple testing correction by Bonferroni adjustment showed a statistically significant improvement in total TWOQ-9 score.”

**Discussion section**

“We acknowledge that our present study has some limitations. First, our study was based on one center and the number of participants was limited.……. Also, there were multiple comparisons in the statistical analysis that might cause statistical significance occurred by chance.”

**3. Whether the improvement of UPDRS score is directly related to the disappearance of H. pylori or alterations in gut microbiota may be addressed in the discussion section.**

**Response:** We appreciate your important comment. There is emerging evidence to support the hypothesis that gut microbiota alteration may be related to the pathogenesis, clinical symptoms, and PD progression. The compositional changes of microbiota may occur after antibiotic usage, smoking, or dietary modifications. In our study, the alterations in microbiota may be occurred after receiving the HP eradiation therapy and might relate to the clinical improvement or L-dopa absorption. Since our study did not aim or provide the baseline type and number of gut microbiota, we could not answer this question. We have addressed this issue in our limitation in the discussion as follows:

“Lastly, benefits seen in our short-term study may not translate into improvements in the longer term, due to variations in lifestyle factors such as dietary habits, the compositional changes of gut microbiota, and other associated factors that might effect on the disease progression or interfere with L-dopa absorption.”

**Reviewer #2: The article describes clearly about the effect of Helicobacter pylori (HP) eradication on the motor and non-motor performance in Parkinson's disease (PD). The authors concluded that HP eradication may increase "on" time and decrease "off" time and also significantly improved the end-of-dose score and GI symptoms including bloating and dysphagia. The article is well written with useful information. However, there are several questions needed to be clarified.**

**1. Is there any difference of all the profiles in patients who failed the eradication therapy? Because PD patients had high placebo effects when receiving the treatment, the data may help clarify the effects of eradication therapy.**

**Response:** We appreciate your comment. We have added the clinical profiles of patients who fail to eradicate therapy in table 3 as your suggestion.

Patients who failed eradication therapy did not demonstrate any benefit after treatment but showed significantly increased daily ‘off’ time (4.9 vs. 4.3 h, p = 0.033) (Table 3).

**Table 3. Clinical symptoms before and after receiving the eradication therapy in *Helicobacter pylori* (HP) eradication failure group (*n* = 5).**

|  | **Before HP eradication** | **After HP eradication** | ***p*** |
| --- | --- | --- | --- |
| Total TWOQ-9 score | 4.8 ± 1.6 | 4.2 ± 2.2 | 0.529 |
| - Tremor | 0.8 ± 0.4 | 0.4 ± 0.5 | 0.178 |
| - Mood changes | 0.2 ± 0.4 | 0.4 ± 0.5 | 0.374 |
| Total GI symptom score | 10.8 ± 4.2 | 11.4 ± 1.3 | 0.763 |
| - Bloating | 1.8 ± 2.2 | 2.6 ± 2.1 | 0.338 |
| Total NMSQ score | 30.0 ± 14.9 | 25.2 ± 16.0 | 0.549 |
| PDQ-8 score | 8.6 ± 3.1 | 9.4 ± 6.4 | 0.759 |
| ‘Off’ UPDRS motor score | 33.0 ± 6.2 | 30.4 ± 8.2 | 0.610 |
| ‘On’ UPDRS motor score | 17.2 ± 7.6 | 12.0 ± 5.0 | 0.127 |
| Time up and go (sec) | 9.1 ± 2.6 | 8.3 ± 1.0 | 0.478 |
| L-Dopa onset time (min) | 23.7 ± 12.4 | 22.0 ± 6.7 | 0.645 |
| L-Dopa peak time (min) | 33.6 ± 12.8 | 28.9 ± 7.3 | 0.248 |
| Daily ‘on’ time (h) | 10.9 ± 2.3 | 9.3 ± 2.3 | 0.191 |
| Daily ‘off’ time (h) | 4.3 ± 2.3 | 4.9 ± 2.3 | **0.033** |
| Dyskinesia (h) | 1.6 ± 2.8 | 1.8 ± 3.3 | 0.374 |

TWOQ-9: Thai version of the 9-item Wearing-off Questionnaire; GI: gastrointestinal; NMSQ: Non-Motor Symptoms Questionnaire; PDQ: Parkinson’s Disease Questionnaire; UPDRS: Unified Parkinson's Disease Rating Scale; L-Dopa: levodopa

Besides, we also added the demographic profiles of patients who fail the eradication therapy in Table 5. Patients who failed the eradication therapy were all male and had a significantly lower intake of vegetables and fruit (1.4 vs. 2.4 servings/day, p < 0.01) as well as a higher rate of smoking (40.0 vs. 0.0%, p < 0.01) compared with the HP eradication group. There was no statistically significant difference in age, age at onset, disease duration, SE-ADL scale, H&Y stage, UPDRS motor score, LED and antiparkinsonian medications between groups (Table 5).

**Table 5. Demographic and clinical baseline status between *Helicobacter pylori* (HP) positive patients who succeeded and failed the eradication therapy.**

|  | **Successful eradication (*n*=17)** | **Failure**  **eradication (*n*=5)** | ***P*** |
| --- | --- | --- | --- |
| Male gender, *n* (%) | 7 (41.2) | 5 (100.0) | **0.020** |
| Age (years) | 61.1 ± 10.6 | 57.4 ± 9.8 | 0.495 |
| Age at onset (years) | 53.3 ± 10.3 | 50.4 ± 9.5 | 0.544 |
| Smoking, n (%) | 0 (0.0) | 2 (40.0) | **0.006** |
| Vegetable/fruit (servings/day) | 2.4 ± 0.6 | 1.4 ± 0.9 | **0.009** |
| Disease duration (years) | 7.6 ± 4.2 | 7.0 ± 5.5 | 0.834 |
| SE-ADL | 77.1 ± 4.7 | 80 ± 0.0 | 0.184 |
| Hoehn & Yahr stage | 2.2 ± 0.6 | 2.2 ± 0.3 | 0.976 |
| ‘Off’ UPDRS motor score | 31.3 ± 10.9 | 33.0 ± 6.2 | 0.664 |
| ‘On’ UPDRS motor score | 13.5 ± 7.5 | 17.2 ± 7.6 | 0.370 |
| Total LED (mg/day) | 774.3 ± 312.7 | 617.9 ± 156.9 | 0.152 |
| - Dopamine agonist, n (%) | 14 (82.4) | 5 (100.0) | 0.312 |
| - COMT inhibitor, n (%) | 9 (52.9) | 1 (20.0) | 0.193 |
| - MAO-B inhibitor, n (%) | 1 (5.9) | 1 (20.0) | 0.334 |
| - Anticholinergic use, n (%) | 6 (35.3) | 1 (20.0) | 0.519 |

SE-ADL: Schwab and England Activities of Daily Living Scale; UPDRS: Unified Parkinson's Disease Rating Scale; LED: Levodopa equivalent dose; COMT: catechol-O-methyltransferase; MAO: monoamine oxidase

**2. Please provide the difference of LED and the percentage of relevant medications of PD patients with successful HP eradication with and without motor fluctuations. Because motor fluctuations may be improved by the adjustment of antiparkinsonian medications, the data may elucidate the effects of the eradication therapy.**

**Response:** Thank you for your comment. All patients in the study had motor fluctuations. All patients who received the HP eradication therapy continued their anti-PD medications and were not allowed to adjust or change their PD medication regimen (mentioned in methods: the HP eradication therapy and outcome assessment section). Therefore, there is no change in LED and the percentage of PD medication before and after receiving HP eradication therapy. We believed that the outcomes in our study were mainly the effects of eradication therapy. However, there is no control of the variations in lifestyle factors such as dietary habits or non-parkinsonian medications that might interfere with L-dopa absorption (mentioned in the discussion).

**3. The International Movement Disorder Society has verified wearing-off questionnaires as WOQ-19 and WOQ-9, which are frequently used. The end-of-dose questionnaire in the article seemed to be similar to the questions of WOQ-9 or WO1-19. Could the authors explain the reasons why using the end-of-dose questionnaire? Please provide the all the profiles of questionnaires and the reference of the questionnaire.**

**Response:** We appreciate your comment and concern. The end-of-dose (EOD) questionnaire that we used in our article/center is similar to the questions of WOQ-9 in Thai language. The questionnaire was developed by Thai Parkinson’s Disease and Movement Disorder Society (Thai PDMDS) and was published in Thai clinical practice guideline for diagnosis and management of PD in 2012. For clarification and understanding, we had edited the name of the questionnaire in our article from “the end-of-dose questionnaire” to "Thai version of the 9-item Wearing-off Questionnaire (TWOQ-9)". We also provided a questionnaire in the supporting file (S1 Study Protocol Thai), data finding in Table 1 and 2, and the article's reference.

**4. It’s very interesting and plausible to know the improvement of bloating symptoms in PD patients receiving eradication therapy. The authors mentioned there was no significant difference of motor scores and NMSQ scores, could the authors provide the evidence and explain the possible mechanism of the improvement of dysphagia symptoms?**

**Response:** Thank you very much for your comment. We have added the possible mechanism in the discussion section as follows:

“Dysphagia typically emerges in PD patients with advanced motor symptoms, and often related to gastroesophageal reflux disease. Eradication of HP in our study did not significantly improve motor scores or reflux symptoms. However, HP positive patients who received eradication treatment demonstrated significant improvement of dysphagia subscore. Also, the post-eradication total GI symptoms, bloating, heartburn, and dysphagia subscores were significantly less than baseline scores of HP negative patients. This might suggest additional effects of eradication therapy, especially from a gastric acid-lowering agent on gastroesophageal reflux disease that might contribute to dysphagia in PD patients. It should be noted that patients with severe dysphagia were excluded from the study. The significant improvement of dysphagia score in our study was minimal and might not significantly be observed in clinical practice.”

6. PLOS authors have the option to publish the peer review history of their article ([what does this mean?](https://journals.plos.org/plosone/s/editorial-and-peer-review-process#loc-peer-review-history)). If published, this will include your full peer review and any attached files.


If you choose “no”, your identity will remain anonymous but your review may still be made public.


**Do you want your identity to be public for this peer review?** For information about this choice, including consent withdrawal, please see our [Privacy Policy](https://www.plos.org/privacy-policy).

Reviewer #1: No

Reviewer #2: No

[NOTE: If reviewer comments were submitted as an attachment file, they will be attached to this email and accessible via the submission site. Please log into your account, locate the manuscript record, and check for the action link "View Attachments". If this link does not appear, there are no attachment files.]

While revising your submission, please upload your figure files to the Preflight Analysis and Conversion Engine (PACE) digital diagnostic tool, <https://pacev2.apexcovantage.com/>. PACE helps ensure that figures meet PLOS requirements. To use PACE, you must first register as a user. Registration is free. Then, login and navigate to the UPLOAD tab, where you will find detailed instructions on how to use the tool. If you encounter any issues or have any questions when using PACE, please email PLOS at [figures@plos.org](mailto:figures@plos.org). Please note that Supporting Information files do not need this step.

**Response:** Thank you very much for your suggestion. We have edited figure files using PACE digital diagnostic tool as your suggestion.

Best regards,
