# Supplementary material for: Helicobacter pylori eradication improves motor fluctuations in advanced Parkinson’s disease patients: A prospective cohort study (HP-PD trial)
Source: PLoS One. 2021 May 4;16(5):e0251042. doi: 10.1371/journal.pone.0251042 (PMC8096108; doi:10.1371/journal.pone.0251042)
Supplement: Attachment — Submitted filename: Response to Reviewers3.docx [file pone.0251042.s006.docx]

Dear Editor,

I am pleased to resubmit the revised version PONE-D-20-33347; “Helicobacter pylori eradication improves motor fluctuations in advanced Parkinson's disease patients: A prospective cohort study (HP-PD trial)”, for publication.

I appreciated the constructive feedback of the editor and reviewers. I have addressed each of their concerns as outlined below.

**Journal Requirements:**

Please review your reference list to ensure that it is complete and correct. If you have cited papers that have been retracted, please include the rationale for doing so in the manuscript text, or remove these references and replace them with relevant current references. Any changes to the reference list should be mentioned in the rebuttal letter that accompanies your revised manuscript. If you need to cite a retracted article, indicate the article’s retracted status in the References list and also include a citation and full reference for the retraction notice.

**Response:** Thank you for your suggestion. We have checked and updated references as your suggestion.

15.	Lolekha P, Kulkantrakorn K. NMSQ application: A new way to access non-motor symptoms in patients with Parkinson’s disease [abstract]. Mov Disord. 2018;33: S526. Available from: <https://www.mdsabstracts.org/abstract/nmsq-application-a-new-way-to-access-non-motor-symptoms-in-patients-with-parkinsons-disease/>

16.	Bhidayasiri R. Clinical Practice Guideline for Diagnosis and Management of Parkinson’s Disease. Bhidayasiri R, editor. Bangkok: Chulalongkorn Centre of Excellence for Parkinson’s Disease & Related Disorders; 2012. Available from: <http://www.chulapd.org/uploads/download_list/2/a-02.pdf>

21.	Mahachai V, Vilaichone RK, Pittayanon R, Rojborwonwitaya J, Leelakusolvong S, Maneerattanaporn M, et al. Helicobacter pylori management in ASEAN: The Bangkok consensus report. Journal of Gastroenterology and Hepatology (Australia). 2018. doi:10.1111/jgh.13911

**Review Comments to the Author:**

**Reviewer #1:** This is an interesting and important study. The authors have responded to the comments from the reviewer.

**Reviewer #2:** The great efforts of the authors to answer the question are appreciated. I think their work may help the clinicians and scientists to uncover the complex mechanism between HP and PD.

**Response:** Thank you very much for your comments.

**Reviewer #3:**

1. **Abstract: There are two percentages mentioned, and it isn't quite clear how these are calculated - 22 patients (55%) and 17 patients (77.3%) - the higher number has the lower percentage, so, to be correct the denominator must be different, but it isn't clear what it is from this text. Could you please clarify, perhaps by stating what the denominator was (22/40 and then 17/22?)?**

**Response:** We appreciate your comment. For clarification, we have edited the abstract as follow:

“A total of 163 PD patients were assessed, of whom 40 were enrolled. Fifty-five percent of the enrolled patients (22/40) had a current HP infection, whereas HP eradication was identified in 17 of 22 (77.3 %) patients who received eradication therapy.”

2. **Study design - pg 3, line 74 - what was the effect size or mean and standard deviation of change in daily on-off time, L-dopa onset or peak time used in the power calculation? What was the difference in groups the study aimed to test? or was it only in those patients who eradicated HP that were of interest? Why were the patients who were HP negative at baseline included?**

**How were the numbers reduced from 163 to 40?** **It is unclear how the various groups were recruited from the text, but it is clearer once Figure 1 is examined - could the text be cleared up?**

**Response:** We appreciate your important comments, and we have addressed each comment as follows:

**- What was the effect size or mean and standard deviation of change in daily on-off time, L-dopa onset or peak time used in the power calculation?**

The effect size we used in our study was set at 0.8. From previous studies, the mean change in L-dopa onset time and total daily on time after eradication therapy were approximately 13 and 106 minutes, respectively. There was no report of the standard deviation of the mean differences. We have revised the paragraph as below:

“The estimated sample size was determined according to the previous studies [11–14] with a level of significance of 5%, the statistical power of 80%, and the effect size of 0.8. The calculated number of HP infected patients needed in this study was 15 patients. Given the prevalence of HP infection in general Thai population of 45% and the possibility of a 15% drop out rate, a total of 40 advanced PD patients were recruited.”

**- What was the difference in groups the study aimed to test? or was it only in those patients who eradicated HP that were of interest?**

The study aimed to compare the clinical effects between before and after receiving HP eradication therapy in advanced PD, so it was mainly focused on PD patients with current HP infection.

**- Why were the patients who were HP negative at baseline included?**

To identify current HP infected patients, we needed to screen the HP status in all advanced PD. We included the HP negative patients in order to identify risk factors or clinical effects that might be associated with HP infection in PD patients. As shown in Table 1, the young age and history of anticholinergic use were risk factors for HP infection. Also, the HP infected patients demonstrated significant delayed L-dopa onset time and tremor during the off period than the non-infected group. These results might support the possible benefit of HP eradication therapy in the later part of our study.

Moreover, there was no significant difference in motor symptoms (UPDRS motor score, daily on-off time, quality of life score) between HP negative patients at baseline and the successful HP eradication therapy group (table 4). These results might suggest that HP eradication is only beneficial in the treatment of motor fluctuations by increased daily on time but not motor symptoms or affects disease progression.

**- How were the numbers reduced from 163 to 40?** **It is unclear how the various groups were recruited from the text, but it is clearer once Figure 1 is examined - could the text be cleared up?**

Thank you for your suggestion, we have revised the recruitment process as follow:

“A total of 163 PD patients were consecutively assessed for eligibility. Of these, 123 patients were excluded, and 40 patients were enrolled in the study (Fig 1).”

3. **pg 9, line 190 - mention multiple testing correction method with statistical methods (as "p<0.05 was considered to be statistically significant") on page 6 isn't the full story. Include the Bonferroni corrected p-values where they were calculated.**

**Response:** Thank you very much for your comment. We did the corrected p-values according to the reviewer’s suggestion in the revised manuscript, and we found that only the total TWOQ-9 score was statistically significant with the corrected *p* = 0.029. We have added the corrected p-values as the following paragraph and provide data of corrected p-values in the supporting table (S1 Table).

“Multiple testing correction by Bonferroni adjustment showed a statistically significant improvement only in total TWOQ- 9 score (corrected *p* = 0.029) (S1 Table).”

4. **Table 4: It needs to be clear that this is baseline HP negative results and after treatment HP eradication results. For the statistically significant results - it may have been that the results were just different at baseline between these two groups of patients - would it have been of interest to see if there was a difference in the later time point data for the two groups, adjusted for the baseline variables? Could be 4 columns of results be presented? Pre and post treatment for those who were HP negative at baseline and those who ended up as HP negative after treatment?**

**Response:** Thank you very much for your suggestion. We have revised Table 4 as below. Patients with HP negative were not reassessed at the 6-week follow-up, so we compared pre-post HP eradication with the HP negative at baseline. From table 4, a significant improvement of the GI symptom scores was observed only in the post-HP eradication. Also, the TWOQ-9 score and L-dopa onset time after the eradication were comparable to the HP negative at baseline.

**Table 4. Clinical symptoms between patients with *Helicobacter pylori* (HP) negative and successful HP eradication groups.**

|  | **HP negative**  **(*n*=18)** | **HP positive at baseline (*n*=17)** | | | |
| --- | --- | --- | --- | --- | --- |
|  |  | **Pre-HP eradication** | ***p^a^*** | **Post-HP eradication** | ***p^a^*** |
| Total TWOQ-9 score | 4.7 ± 2.0 | 6.0 ± 1.3 | **0.028** | 4.4 ± 1.7 | 0.625 |
| Total GI symptom score | 15.4 ± 8.1 | 12.8 ± 7.3 | 0.335 | 8.1 ± 4.5 | **0.003** |
| - Heartburn | 2.4 ± 2.8 | 1.2 ± 2.8 | 0.211 | 0.6 ± 1.4 | **0.024** |
| - Bloating | 3.3 ± 2.7 | 2.9 ± 3.2 | 0.740 | 1.1 ± 1.6 | **0.007** |
| - Nausea | 0.6 ± 1.5 | 0.2 ± 0.5 | 0.267 | 0.2 ± 0.5 | 0.267 |
| - Vomiting | 0.1 ± 0.5 | 0.1 ± 0.5 | 0.968 | 0.1 ± 0.5 | 0.968 |
| - Abdominal pain | 1.6 ± 2.5 | 1.8 ± 2.6 | 0.760 | 0.9 ± 2.2 | 0.404 |
| - Diarrhea | 0.5 ± 1.2 | 0.4 ± 1.1 | 0.819 | 0.0 ± 0.0 | 0.096 |
| - Constipation | 6.9 ± 3.5 | 6.2 ± 3.5 | 0.516 | 5.2 ± 2.8 | 0.115 |
| Total NMSQ score | 37.9 ± 20.9 | 33.4 ± 13.3 | 0.544 | 29.3 ± 14.1 | 0.173 |
| - Dysphagia | 1.3 ± 1.3 | 0.7 ± 1.0 | 0.124 | 0.3 ± 0.6 | **0.008** |
| PDQ-8 score | 9.2 ± 6.8 | 10.9 ± 5.3 | 0.409 | 8.6 ± 5.2 | 0.759 |
| ‘Off’ UPDRS motor score | 29.8 ± 14.8 | 29.9 ± 9.6 | 0.740 | 26.0 ± 10.0 | 0.388 |
| ‘On’ UPDRS motor score | 15.1 ± 11.3 | 12.7 ± 7.0 | 0.618 | 10.6 ± 5.0 | 0.146 |
| Time up and go (sec) | 12.1 ± 6.3 | 11.8 ± 8.5 | 0.817 | 9.8 ±2.8 | 0.289 |
| L-Dopa onset time (min) | 15.4 ± 10.0 | 21.2 ± 6.9 | **0.041** | 19.5 ± 9.6 | 0.229 |
| L-Dopa peak time (min) | 34.5 ± 10.5 | 35.7 ± 7.9 | 0.669 | 34.9 ± 13.8 | 0.979 |
| Daily ‘on’ time (h) | 12.0 ± 2.4 | 10.9 ± 1.9 | 0.140 | 11.8 ± 2.0 | 0.781 |
| Daily ‘off’ time (h) | 3.9 ± 2.2 | 4.7 ± 1.5 | 0.181 | 4.0 ± 2.1 | 0.819 |
| Dyskinesia (h) | 1.5 ± 2.3 | 1.1 ± 1.6 | 0.630 | 1.3 ± 1.8 | 0.802 |

^a^ Compared to HP negative group.
TWOQ-9: Thai version of the 9-item Wearing-off Questionnaire; GI: gastrointestinal; NMSQ: Non-Motor Symptoms Questionnaire; PDQ: Parkinson’s Disease Questionnaire; UPDRS: Unified Parkinson's Disease Rating Scale; L-Dopa: levodopa
Statistically significant *p*-values are in bold

Best regards,
